# Case report: Balloon compression for cervical chyle leakage post neck dissection

**DOI:** 10.3389/fsurg.2022.1019425

**Published:** 2022-09-23

**Authors:** Zhaoming Ding, Mengshi Chen, Rui Pang, Ruinan Sheng, Xuesong Zhao, Chunlei Nie

**Affiliations:** Department of Thyroid Surgery, Harbin Medical University Cancer Hospital, Harbin, China

**Keywords:** chyle leakage, thyroid cancer, neck dissection, balloon compression, case report

## Abstract

Postoperative chyle leakage (CL) is a rare but severe complication after neck dissection, and most patients with this complication can be treated conservatively. However, in patients with high-flow leakage, efficient and well-tolerated conservative treatment options are still lacking, and the treatments can be complicated. In this study, we report a case with CL of 1100 ml/day after neck dissection that was successfully treated by balloon compression.

## Introduction

Chyle leakage (CL) is a rare but severe complication of thyroid surgery with neck dissection, and the main cause of CL may be trauma to the thoracic duct and its main distribution (1). Prolonged CL can cause severe malnutrition, psychological depression, or even mortality. Prompt identification and treatment of CL are essential for optimal surgical outcomes (2).

Currently, there is no standardized treatment for the management of CL (3); usually, the first line of treatment is conservative management, such as a modified diet, drainage of effusion, pressure dressings, and the administration of octreotide and etilefrine (4), which are useful for most cases. However, high output fistulas (>1 L/day) or prolonged drainage with a low CL volume (a duration >7 to 14 days) will often respond unsatisfactorily to conservative management alone and require surgical intervention (5, 6). Herein, we report a case of a patient with high-volume cervical CL of 1100 ml/day who was treated successfully by balloon compression, and the patient successfully avoided reoperation. This study was reported in agreement with principles of the CARE guidelines (7).

## Case presentation

A 70-year-old man was admitted to our hospital due to the presence of a thyroid tumor and lymphadenopathy in the left lower neck diagnosed on US and CT. Fine-needle biopsy was used to make the diagnosis of PTC, clinically T1N1bM0. After discussing treatment options with the patient, total thyroidectomy with central and left lateral neck dissection was performed. During the operation, multiple metastatic lymph nodes with a maximum diameter of 3 cm in level IV were cleared; on the premise of completing oncological resection, the trunk of the thoracic duct was preserved. In the thyroid cavity, a single drain was placed; in the left neck cavity, two drains were placed and drained out from the anterior and posterior edges of the sternocleidomastoid muscle separately. Histopathological examination revealed that a total of 43 lymph nodes were removed in the left lateral neck compartment, and 20 of them were found to have tumor metastasis. Among them, in level IV, the number of metastatic/total lymph nodes was 8/10.

**Figure 1:**
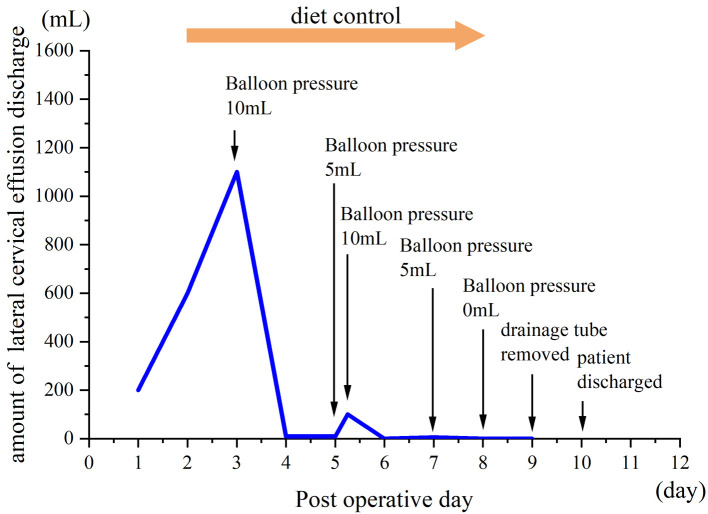
Postoperative progress and amount of discharge from lateral cervical drainage tube.

**Figure 2:**
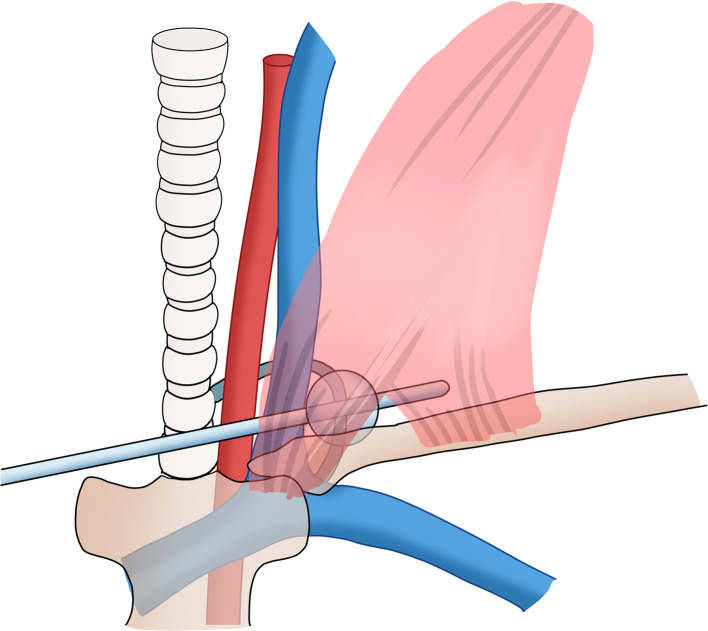
Schematic drawings of the balloon compression maneuver. Copyright © [2022] [Zhaoming Ding]: reproduced with permission.

Postoperatively (Figure 1), on the first postoperative day (POD1), the fluid in the lateral neck cavity drain was 200 ml/d, which was bloody in color, and this is common after surgery. On the second postoperative day (POD2), this drain produced 600 ml of milky fluid. The volume obtained from the drain increased to 1100 ml per day on POD3, even after the patient was treated with fasting, total parenteral nutrition and pressure dressings with elastic bandages. Because of the large amount of chyle, we speculated that it would be unlikely that conservative treatment would be effective, and the massive lymph loss may also be associated with mortality. Therefore, we decided to take some active interventions and designed a balloon-compression maneuver to improve the pressure dressing therapy. We removed the lateral neck drainage tube of the anterior edge of sternocleidomastoid, placed a size 14 sterile urinary catheter and retraced and connected a negative pressure drainage. The balloon was placed between the internal jugular vein and the sternal head of sternocleidomastoid, which was located by palpation. Then, 10 ml normal saline was injected into the balloon, creating local pressure at the possible lymphatic damage area at the venous angle (Figure 2). When the patient's neck moved, the balloon tended to move inward rather than fall out; therefore, we fixed the drainage tube on the chest wall skin with adhesive tape to ensure that the position of the balloon would be fixed. After balloon compression, the amount of discharge from the cervical drainage tube decreased immediately to 10 ml/24 h. On this day, we also removed the drain in the thyroid cavity, and the drain had a total drainage of 60 ml, which was hemoserous.

On POD5, we tentatively reduced the balloon volume to 5 ml, but the amount of discharge increased to 100 ml/6 h. We adjusted the balloon volume to 10 ml again, and the steady chyle leakage stopped. On POD7, we reduced the balloon volume to 5 ml, and the amount of discharge did not increase. The patient resumed a normal diet on POD8; during this time, there was still no volume of fluid that was collected. On POD9, the drainage tube was removed, the patient was discharged from the hospital on POD10, and no neck swelling was reported. No cervical chyle-related symptoms were found after one month of follow-up.

## Discussion

Chyle leakage (CL) following neck dissection for thyroid carcinoma is infrequent but represents a serious complication. Several studies have reported chyle leakage incidences of 0.6%–1.4% after CND and 4.5%- 8.3% after lateral neck dissection (8–11). The incidence varies according to the surgical extent, and it more commonly occurs when extranodal extension is involved around the level IV compartment (12). According to the results of a previous study, a CL volume of 1 L/day is often used as a cutoff value to classify CL volumes as low and high (6). High-volume CL is a clinically inevitable and severe problem that can cause hypovolemia, malnutrition, electrolyte disturbances, metabolic imbalance, immunosuppression, dehydration, poor wound healing and prolonged length of hospital stay (13). Early identification and appropriate management of CL are imperative for optimal surgical outcomes. Meticulous protection of the thoracic duct during surgery is crucial to prevent CL, but the variation in the termination and fragility of the thoracic duct renders it difficult to avoid completely, even when the operation is performed by an experienced surgeon (14–17).

The management of CL that is detected postoperatively is challenging, and conservative management, including NPO with total parenteral nutrition, administration of medium-chain triglycerides and octreotide, repeated aspiration, negative pressure drainage, and pressure dressings (4), are the first-line treatment approaches, and these treatments are performed to promote spontaneous fistula closure by diminishing the chyle flow. However, if conservative therapy fails, invasive interventions are recommended, including lymphangiographic embolization of the thoracic duct, thoracoscopic thoracic duct ligation, and the use of local rotational muscle flaps to obliterate the leakage sites (17–19).

To date, there are no consensus guidelines on the timing of surgical exploration, and it is usually considered that surgical intervention should be decided upon within the first 4–5 days of CL when the patient does not develop a prompt response to medical management (20). Meanwhile, the surgical approach will cause patients to suffer more and is associated with an increase in complications. We aimed to find a new conservative treatment to cure CL postoperatively.

Pressure dressing is a simple, initial and effective approach that is applied in surgery to deal with hemorrhage or leakage. In the treatment of CL, pressure dressings are used to compress the leaking chyle vessel and allow time for spontaneous closure (11, 20). However, traditional pressure dressings are ineffective for most patients when we use thick dressings with elastic bandages to put pressure on the lower neck. Since the thoracic duct is located deep in the venous angle and due to the occlusion of the anterior clavicle and sternocleidomastoid muscle, it is difficult to place an adequate amount of pressure on the precise point of leakage with only external pressure. Moreover, excessive pressure causes significant patient discomfort and affects skin flap viability (19). Interestingly, a previous study introduced a simple, finger-based pressing maneuver to stop CL, although it may be more challenging to widely adopt because of the labor required by the person carrying out continuous pressing; it is effective and comfortable compared with the traditional methods (21). In this case, we applied a new method based on balloon pressure. We used the balloon of a urinary catheter to compress the CL at the angle of the vein and accurately achieved appropriate and mild compression on the crucial pressing point.

We confirmed that the integrity of the thoracic duct trunk during the operation, as the TD route and terminations demonstrate many variations. We considered that this CL may be due to injury of an anatomical variation of TD terminations or its tributaries. Since the patient presented with high-output CL and failed to respond to conservative management, to avoid the burden of reoperation, we used a balloon to compress the venous horn to reduce the lymph exudation and provide conditions for spontaneous healing. To avoid the potential risk of chylothorax caused by excessive compression, another negative pressure drainage tube was kept indwelling until it was finally removed with the urinary catheter. In this case, our prompt treatment reduced the loss of lymph, the patient avoided reoperation, and the treatment shortened the hospital stay. To the best of our knowledge, this is the first and only case of CL treatment by balloon pressure. This treatment is simple, safe and well tolerated by patients, and the rapid reduction of drainage volume also increases the patient confidence and reduces anxiety.

This single case report had some limitations. First, we located the balloon by palpation, but this method is not accurate enough. It might be better to use guided ultrasound to locate the exact position of the damage and place the balloon. Second, we speculated where the point of leakage was located through clinical experience and intraoperative findings, and it might be better to determine the precise point of leakage before balloon pressure. Third, we overlooked the potential thrombotic risk of balloon compression on the vein; fortunately, the patient did not develop thrombosis-related complications, possibly because the mild compression did not completely compress the internal jugular vein. Ultrasound should be performed after balloon compression to confirm that the internal jugular vein is still unobstructed, and daily ultrasound should be used to confirm that there is no thrombosis in the vein. Moreover, we are not sure whether anticoagulation therapy should be used. Fourth, this was only a single case. Although the patient, who had CL of 1,100 ml/day, was managed successfully by balloon pressure, without surgical intervention, firm conclusions regarding the safety, feasibility, and efficacy of balloon pressure require further investigation in a prospective series. A suitable anatomical model to study the compression effect of pressure on lymphatic vessels is also required.

## Conclusion

In conclusion, the results of this case suggest that high-volume CL post neck dissection might be managed conservatively with balloon pressure. This technique provides a considered conservative option for treating CL.

## Data Availability

The original contributions presented in the study are included in the article/Supplementary Material, further inquiries can be directed to the corresponding author/s.

## References

[1] Park I Her N Choe JH Kim JS Kim JH. Management of chyle leakage after thyroidectomy, cervical lymph node dissection, in patients with thyroid cancer. Head Neck. (2018) 40(1):7–15. 10.1002/hed.24852 29120521

[2] Delaney SW Shi H Shokrani A Sinha UK. Management of chyle leak after head and neck surgery: review of current treatment strategies. Int J Otolaryngol. (2017) 2017:8362874. 10.1155/2017/8362874 28203252 PMC5288539

[3] Molena E King E Davies-Husband C. Octreotide versus oral dietary modification for the treatment of chylous fistula following neck dissection: a systematic review and meta-analysis. Clin Otolaryngol. (2021) 46(3):474–84. 10.1111/coa.13700 33342047

[4] Chan JY Wong EW Ng SK van Hasselt CA Vlantis AC. Conservative management of postoperative chylous fistula with octreotide and peripheral total parenteral nutrition. Ear Nose Throat J. (2017) 96(7):264–7. 10.1177/014556131709600720 28719710

[5] Lv S Wang Q Zhao W Han L Wang Q Batchu N A review of the postoperative lymphatic leakage. Oncotarget. (2017 Apr) 8(40):69062–75. 10.18632/oncotarget.17297 28978181 PMC5620321

[6] Chang GH Lee CY Tsai YT Fang CC Fang KH Tsai MS Strategic approach to massive chylous leakage after neck dissection. Healthcare (Basel. (2021) 9(4):379. 10.3390/healthcare9040379 33807397 PMC8067092

[7] Riley DS Barber MS Kienle GS Aronson JK von Schoen-Angerer T Tugwell P CARE Guidelines for case reports: explanation and elaboration document. J Clin Epidemiol. (2017) 89:218–35. 10.1016/j.jclinepi.2017.04.026 28529185

[8] Kupferman ME Patterson DM Mandel SJ LiVolsi V Weber RS. Safety of modified radical neck dissection for differentiated thyroid carcinoma. Laryngoscope. (2004) 114(3):403–6. 10.1097/00005537-200403000-00002 15091209

[9] Roh JL Yoon YH Park CI. Chyle leakage in patients undergoing thyroidectomy plus central neck dissection for differentiated papillary thyroid carcinoma. Ann Surg Oncol. (2008) 15(9):2576–80. 10.1245/s10434-008-0017-9 18592317

[10] Lee YS Kim BW Chang HS Park CS. Factors predisposing to chyle leakage following thyroid cancer surgery without lateral neck dissection. Head Neck. (2013) 35(8):1149–52. 10.1002/hed.23104 23019144

[11] Lee YS Nam KH Chung WY Chang HS Park CS. Postoperative complications of thyroid cancer in a single center experience. J Korean Med Sci. (2010) 25(4):541–5. 10.3346/jkms.2010.25.4.541 20357995 PMC2844597

[12] Dunlap Q Bridges M Nelson K King D Stack BC Jr Vural E Predictors for postoperative chyle leak following neck dissection, a technique-based comparison. Otolaryngol Head Neck Surg. (2021) 165(5):667–72. 10.1177/0194599821993815 33687279

[13] Ilczyszyn A Ridha H Durrani AJ. Management of chyle leak post neck dissection: a case report and literature review. J Plast Reconstr Aesthet Surg. (2011) 64(9):e223–30. 10.1016/j.bjps.2010.12.018 21296632

[14] Campisi CC Boccardo F Piazza C Campisi C. Evolution of chylous fistula management after neck dissection. Curr Opin Otolaryngol Head Neck Surg. (2013) 21(2):150–6. 10.1097/MOO.0b013e32835e9d97 23449286

[15] Phang K Bowman M Phillips A Windsor J. Review of thoracic duct anatomical variations and clinical implications. Clin Anat. (2014) 27(4):637–44. 10.1002/ca.22337 24302465

[16] Hematti H Mehran RJ. Anatomy of the thoracic duct. Thorac Surg Clin. (2011) 21(2):229–38. 10.1016/j.thorsurg.2011.01.002 21477773

[17] Alejandre-Lafont E Krompiec C Rau WS Krombach GA. Effectiveness of therapeutic lymphography on lymphatic leakage. Acta Radiol. (2011) 52(3):305–11. 10.1258/ar.2010.090356 21498367

[18] Teksoz S Ersen E Arikan AE Ferahman S Kaynak K Dionigi G Single port thoracoscopic treatment of thoracic duct injury after thyroidectomy with neck dissection. Gland Surg. (2017) 6(5):598–601. 10.21037/gs.2017.07.14 29142855 PMC5676156

[19] de Gier HH Balm AJ Bruning PF Gregor RT Hilgers FJ. Systematic approach to the treatment of chylous leakage after neck dissection. Head Neck. (1996) 18(4):347–51. 10.1002/(SICI)1097-0347(199607/08)18:4<347::AID-HED6>3.0.CO;2-Y 8780946

[20] Nussenbaum B Liu JH Sinard RJ. Systematic management of chyle fistula: the southwestern experience and review of the literature. Otolaryngol Head Neck Surg. (2000) 122(1):31–8. 10.1016/S0194-5998(00)70140-9 10629479

[21] Xiang D Liu Z Yang T Bai B Zhang J Wang C Finger-pressing: a simple and efficient way to stop chyle leak post neck dissection. Endocrine. (2020) 67(2):374–8. 10.1007/s12020-019-02119-0 31673955

